# Diagnostic accuracy of anti-phospholipase A2 receptor (PLA2R) antibodies in idiopathic membranous nephropathy: an Italian experience

**DOI:** 10.1007/s40620-020-00888-w

**Published:** 2020-10-29

**Authors:** Brunetta Porcelli, Andrea Guarnieri, Fabio Ferretti, Guido Garosi, Lucia Terzuoli, Francesca Cinci, Antonella Tabucchi, Marilina Tampoia, Letizia Abbracciavento, Chiara Villani, Gaia Deleonardi, Ana Gabriela Grondona, Marcello Mazzolini, Gaetano La Manna, Marisa Santostefano, Maria Infantino, Mariangela Manfredi, Giuseppe Spatoliatore, Alberto Rosati, Chiara Somma, Nicola Bizzaro

**Affiliations:** 1 Dipartimento Biotecnologie Mediche, Sezione Biochimica, Università degli Studi di Siena, Polo Scientifico Universitario San Miniato, Via Alcide De Gasperi 2, 53100 Siena, Italy; 2 UOC Laboratorio Patologia Clinica, Policlinico S. Maria Alle Scotte, AOU Senese, Siena, Italy; 3 UOC Nefrologia, Dialisi e Trapianti, Policlinico S. Maria alle Scotte, AOU Senese, Siena, Italy; 4 Dipartimento Scienze Mediche, Chirurgiche e Neuroscienze, Università degli Studi di Siena, Siena, Italy; 5 UOC Patologia Clinica Universitaria, Dipartimento Scienze Biomediche e Oncologia Umana, Azienda Ospedaliero-Universitaria, Policlinico di Bari, Bari, Italy; 6 UOC Nefrologia Universitaria, Dipartimento dell’Emergenza e dei Trapianti d’Organo, Azienda Ospedaliero-Universitaria, Policlinico di Bari, Bari, Italy; 7 Laboratorio Unico Metropolitano, Ospedale Maggiore, Bologna, Italy; 8 Dipartimento Scienze Mediche e Chirurgiche, Università degli Studi di Bologna, Bologna, Italy; 9 Dipartimento Medicina Specialistica, Diagnostica e Sperimentale, Università degli Studi di Bologna, Bologna, Italy; 10 UO Nefrologia, Dialisi e Trapianto, Policlinico Universitario S. Orsola-Malpighi, Bologna, Italy; 11 U.O. Nefrologia, Dialisi e Ipertensione, Azienda Ospedaliera-Universitaria Sant’Orsola, Bologna, Italy; 12 Laboratorio Immunologia e Allergologia, Dipartimento di Medicina di Laboratorio, Ospedale San Giovanni di Dio, AUSL Toscana Centro, Firenze, Italy; 13 SOC Nefrologia e Dialisi, Ospedale San Giovanni di Dio, AUSL Toscana Centro, Firenze, Italy; 14 SOC Nefrologia e Dialisi, Ospedale Santa Maria Annunziata, AUSL Toscana Centro, Firenze, Italy; 15 Laboratorio di Patologia Clinica, Ospedale San Antonio, Azienda Sanitaria Universitaria Integrata di Udine, Tolmezzo, Italy

**Keywords:** Anti-phospholipase A2 receptor (PLA2R) autoantibodies, Membranous nephropathy, Cut-off value, Enzyme-linked immunosorbent assay (ELISA)

## Abstract

**Background:**

Autoantibodies against-phospholipase A2 receptor (PLA2R) are specific markers of idiopathic membranous nephropathy (iMN). Enzyme-linked immunosorbent assay (ELISA) is becoming the preferred method in many laboratories for the determination of anti-PLA2R antibodies, because it provides quantitative results, and is not prone to subjective interpretation, as is the case with indirect immunofluorescence assay.

**Methods:**

The purpose of our study was to determine the diagnostic performance of serum PLA2R antibodies detected by commercially available ELISA in a large Italian multicenter cohort of patients with biopsy-proven iMN and in patients with other renal diseases, with special focus on evaluating the optimal cut-off value to discriminate positive and negative results. A total of 495 consecutive patients were recruited. Renal biopsies were performed in all patients, and blood samples were taken before the initiation of immunosuppressive treatment.

**Results:**

According to the clinical diagnosis and to kidney biopsy, 126 patients were diagnosed with iMN and 369 had other non-membranous nephropathies. Anti-PLA2R autoantibodies were detected using a commercial anti-PLA2R ELISA. At a cut-off value of 20 relative units (RU)/ml indicated by the manufacturer for positive classification, sensitivity was 61.1% and specificity 99.7%. At a cut-off value of 14 RU/ml indicated by the manufacturer for borderline results, sensitivity was 63.5% and specificity remained the same (99.7%). At a cut-off of 2.7 RU/ml, selected as the optimal cut-off on the basis of ROC curve analysis, sensitivity was 83.3% and specificity 95.1%. The best overall efficiency of the test was observed at 2.7 RU/ml; however, the highest positive likelihood ratio and diagnostic odds ratio were achieved at 14 RU/ml. A cut-off threshold higher than 14 RU/ml or lower than 2.7 RU/ml entailed worse test performance.

**Conclusion:**

Depending on the clinical use (early diagnosis or as a support to confirm clinical diagnosis), nephrologists may take advantage of this evidence by choosing the most convenient cut-off. However, renal biopsy remains mandatory for the definitive diagnosis of iMN and for the assessment of disease severity.

## Introduction

Membranous nephropathy (MN) is a leading cause of nephrotic syndrome in adults. MN can be either idiopathic (iMN) or secondary (sMN) to various clinical conditions, including systemic autoimmune diseases, infections, neoplasia and drug intoxication [1–3]. In 2009, Beck et al. [4] showed that antibodies in serum samples from subjects with iMN specifically identified a 185-kDa glycoprotein in non-reduced glomerular extract by western blotting. Mass spectrometry of the reactive protein band detected the phospholipase A2 receptor (PLA2R), a membrane glycoprotein located on the normal renal glomerular podocytes and present in kidney immune deposits, indicating that PLA2R is a major antigen in this disease. This finding led to the subsequent development of anti-PLA2R antibody tests as an aid in the differential diagnosis of iMN from sMN and other nephropathies displaying similar clinical features [4–6]. In addition, serial measurement of anti-PLA2R antibodies may prove useful for prognosis and in guiding treatment in iMN patients [7].

Recent meta-analyses showed a prevalence of serum PLA2R antibodies in iMN patients ranging between 30 and 89% depending mainly on the ethnic population and on the detection method [8–11]. Anti-PLA2R antibodies can be detected by western blot (WB), and by commercial methods such as indirect immunofluorescence assay (IFI) or enzyme-linked immunosorbent assay (ELISA), all displaying high diagnostic specificity and high concordance [12–14]. However, ELISA is becoming the most widely used method to detect anti-PLA2R antibodies in clinical practice due to its advantages of offering quantitative results and suitability for automation. Quantitative results are important in monitoring disease progression and response to therapy [15–17].

Most studies have been conducted in Asia (especially in China) compared to the few studies conducted in western countries. Therefore, the purpose of our study was to determine the diagnostic performance of serum PLA2R antibodies detected by commercially available ELISA in a large Italian multicenter cohort of patients with biopsy-proven iMN and in patients with other renal diseases, with special focus on evaluating the optimal cut-off value to discriminate positive and negative results. We also analyzed biomarkers of disease activity in anti-PLA2R autoantibody-positive and -negative patients with iMN.

## Methods

### Patients

A total of 495 consecutive patients, all Caucasian, admitted to the Nephrology Units (Siena University Hospital, Bari University Hospital, Bologna University Hospital, San Giovanni di Dio Hospital and Santa Maria Annunziata Hospital, Florence, Italy) between January 2016 and January 2018 with a request for anti-PLA2R antibody testing, were enrolled in this study. Renal biopsies and blood and urine tests were performed at baseline, before the initiation of immunosuppressive treatment, in all patients. According to the clinical diagnosis and to kidney biopsy, which were adopted as the diagnostic gold standard for this study, 126 patients were diagnosed with iMN (mean age, 58.78 ± 16.97; F/M ratio, 0.48) and 369 (mean age 53.03 ± 16.92; F/M ratio, 0.56) had other non-membranous nephropathies (Table 1).

**Table 1:** Specific renal disease in the control group of patients

Disease	No. patients (%)
IgA-nephropathy	75 (20.3%)
Focal and segmental glomerular sclerosis	83 (22.5%)
Minimal change disease	24 (6.5%)
Systemic lupus erythematosus	19 (5.1%)
Diabetes	28 (7.6%)
ANCA-positive vasculitis	27 (7.3%)
Other clinical conditions	113 (30.6%)

Serum samples, collected at the sites where the patients were diagnosed were tested within 48 h or frozen at − 20 °C until testing.

The study was conducted in accordance with the ethical standards as formulated in the Helsinki Declaration and with the Italian legislation (Authorization of the Privacy Guarantor No. 9, December 12th, 2013).

### Anti-PLA2R measurement

Anti-PLA2R autoantibodies were detected using a commercial anti-PLA2R ELISA (Euroimmun, Luebeck, Germany) based on purified human recombinant PLA2R antigen; the use of a standard curve consisting of five calibrators (2, 20, 100, 500, and 1500 relative units (RU)/ml) and inclusion of a blank serum as zero RU/ml calibrator, allows to provide continuous quantitative results for anti-PLA2R autoantibody concentration [18]. Samples were run in duplicate. According to the manufacturer’s recommendations, values ≥ 20 RU/ml are considered positive, while values between 14 and 19 RU/ml are borderline, and values < 14 RU/ml are considered negative. In this study, we evaluated anti-PLA2R antibody ELISA results at the two different cut-offs suggested by the manufacturer for negative and positive classification (14 and 20 RU/ml, respectively), and at cut-off values obtained by receiver operating characteristic (ROC) curves.

### Other biochemical parameters

Serum creatinine, serum albumin and 24-h proteinuria were measured in the recruiting centers at the time of diagnosis. The estimated glomerular filtration rate (eGFR) was calculated from serum creatinine by the Chronic Kidney Disease Epidemiology Collaboration (CKD-EPI) formula adjusted for sex and ethnic origin [19].

### Statistical analysis

Sensitivity (in 126 patients with iMN), specificity (in 369 patients with other non-membranous nephropathies), diagnostic efficiency (the overall probability that a patient is correctly classified), positive and negative predictive values (PPV and NPV), likelihood ratios (LR+, LR−) and diagnostic odds ratio (DOR) with 95% confidence interval (95%CI) of anti-PLA2R ELISA results were calculated. ROC curve analysis was performed for optimal cut-off positioning, and the area under the curve (AUC) with 95% CI was determined. MedCalc software (Mariakerke, Belgium) was used for ROC curve analysis.

The Kolmogorov–Smirnov test for normality was performed on quantitative variables (anti-PLA2R, serum creatinine, serum albumin, 24 h proteinuria and eGFR). As a consequence of the violation of normality, the non-parametric Mann–Whitney test was used to assess the significance of the difference between groups of patients. Statistical analyses were performed with SPSS-IBM v23 and the level of significance was set at p < 0.05.

## Results

**Fig. 1:**
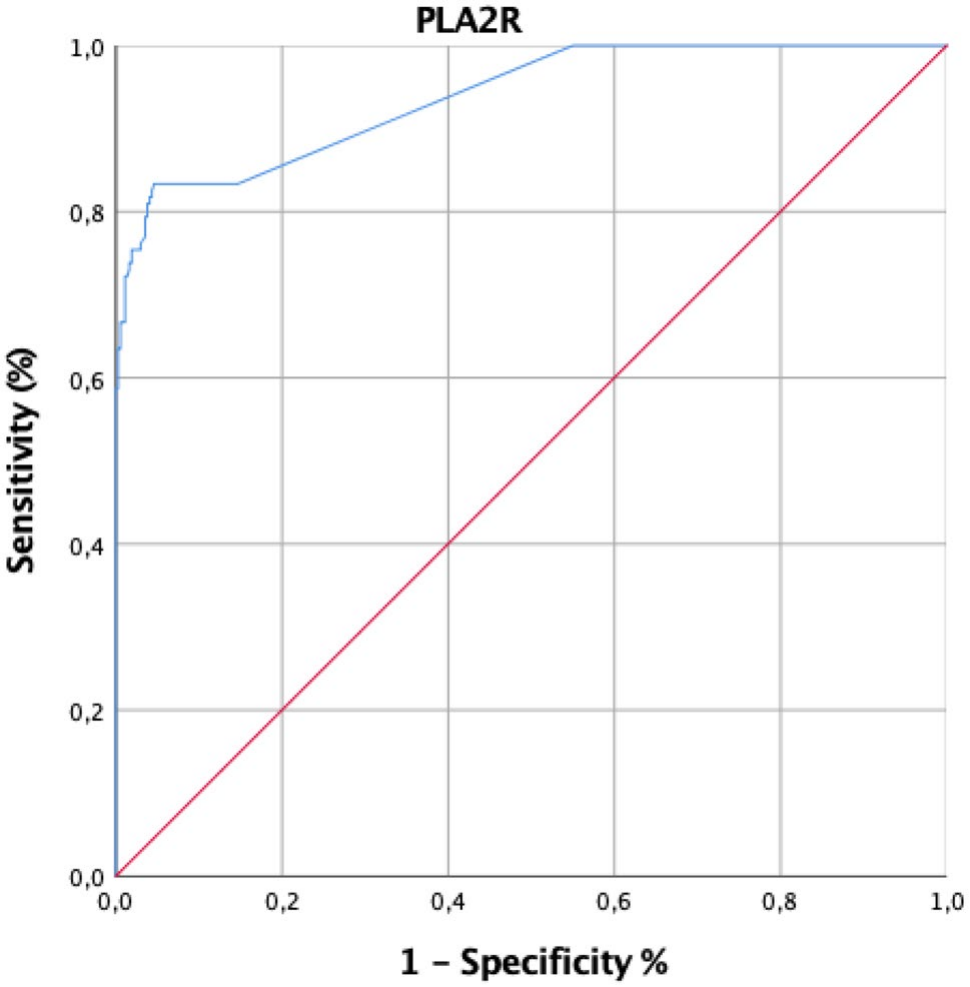
ROC curve of anti-PLA2R for the identification of patients with iMN

The demographic and biochemical characteristics of iMN patients at baseline are described in Table 2. Performance characteristics of the PLA2R ELISA at different cut-off values (2, 2.7, 14, 20 and 40 RU/ml) are described in Table 3. At a cut-off value of 20 RU/ml indicated by the manufacturer for positive classification, sensitivity was 61.1% and specificity 99.7%. At a cut-off value of 14 RU/ml indicated by the manufacturer for borderline results, sensitivity was 63.5% and specificity remained the same (99.7%). At a cut-off of 2.7 RU/ml, selected as the optimal cut-off on the basis of ROC curve analysis, one hundred-five/126 of patients with iMN (83.3%; 95% CI 76.6–89.9) and 18/369 (4.9%; 95% CI 2.8–7.0%) with non-membranous nephropathies were positive for anti-PLA2R antibodies (all but one at a value < 10 RU/ml). Sensitivity was 83.3% and specificity 95.1% (Fig. 1). The value of the AUC was 0.938 (95% CI 0.912–0.963). However, the highest positive likelihood ratio and diagnostic odds ratio were achieved at 14 RU/ml. A higher cut-off threshold than 14 RU/ml or a lower one than 2.7 RU/ml entailed worse test performance.

**Table 2:** Baseline characteristics of patients with iMN

	Patients (n = 126)
Sex (male/female)	85 (67.5%)/41 (32.5%)
Age at diagnosis (years)^a^	58.78 ± 16.97
Serum creatinine (mg/dl)^b^	1.01 (0.84–1.66)
Serum albumin (g/dl)^b^	2.20 (1.80–2.60)
Proteinuria (g/24 h)^b^	6.70 (4.00–9.50)
eGFR (ml/min/1.73 m^2^)^b^	74.00 (38.00–99.00)
Anti-PLA2R (RU/ml)^b^	63.70 (12.50–214.80)

*IMN* idiopathic membranous nephropathy; *eGFR* estimated glomerular filtration rate; *PLA2R:* phospholipase A2 receptor

^a^Mean ± SD

^b^Median (interquartile range)

**Table 3:** Performance characteristics (with 95% confidence intervals) of anti-PLA2R ELISA at different cut-off values as determined by ROC curve analysis

Cutoff value	2.0 RU/ml	2.7 RU/ml	14 RU/ml	20 RU/ml	40 RU/ml
Sensitivity	100% (97.1–100)	83.3% (76.8–89.8)	63.4% (55.4–71.8)	61.1% (52.6–69.6)	51.5% (42.5–60.5)
Specificity	45.0% (39.8–50.2)	94.5% (92.9–97.3)	99.7% (98.5–99.9)	99.7% (99.2–100.3)	100% (99.0–100)
Efficiency	59.0% (54.5–63.3)	91.7% (88.9–93.9)	90.5% (87.5–92.9)	89.9% (86.9–92.4)	87.6% (84.4–90.4)
PPV	38.3% (33.0–43.8)	84.0% (76.3–89.9)	98.7% (93.3–99.9)	98.7% (93.0–99.9)	100% (94.4–100)
NPV	100% (97.8–100)	94.3% (91.4–96.4)	88.9% (85.4–91.7)	88.2% (84.7–91.1)	85.8% (82.1–88.9)
LR+	1.8	15.3	234	225	∞
LR−	0.01	5.6	2.7	2.5	2.0
DOR	69	87.2	640	578	787

PPV, positive predictive value; NPV, negative predictive value; LR+, positive likelihood ratio; LR−, negative likelihood ratio; DOR, diagnostic odds ratio

**Fig. 2:**
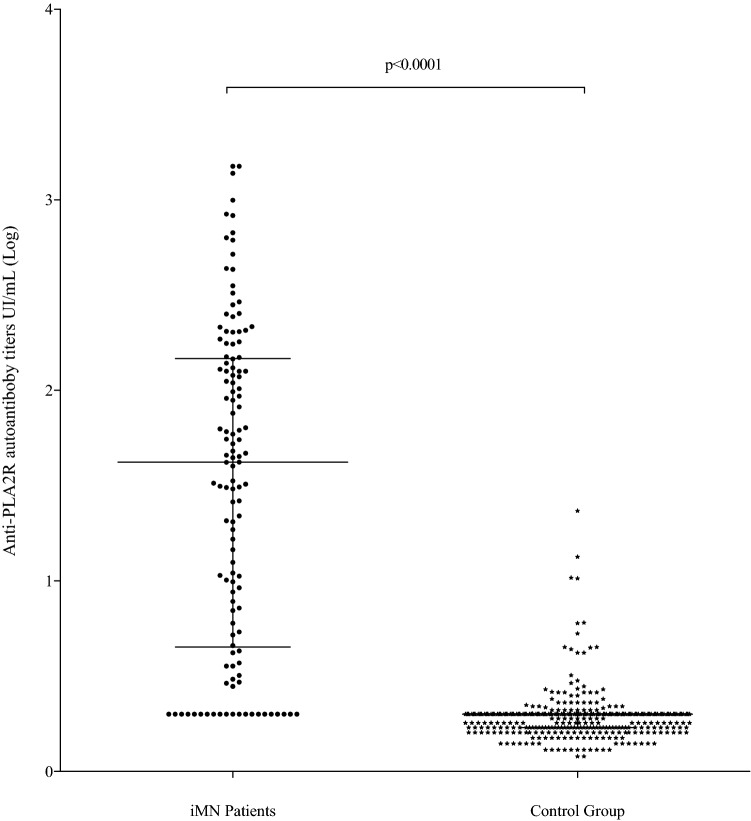
Distribution of anti-PLA2R autoantibody levels in iMN patients and in the control group

In patients with iMN, antibody levels varied between 2.00 RU/ml and 1,500 RU/ml and the median antibody level was 42 RU/ml (interquartile range [IQR], 4.50–146.8 RU/ml), significantly higher than in patients with other nephropathies (range, 1.20–23.3 RU/ml; median concentration 2.00 RU/ml [IQR 1.70–2.00 RU/ml]; *p* < 0.0001) (Fig. 2). Stratification of cases according to histological grading of renal biopsy did not correlate with anti-PLA2R antibody levels (data not shown).

Demographic features and biomarkers of disease activity in anti-PLA2R autoantibody-positive and -negative patients with iMN are reported in Table 4. Age was higher in anti-PLA2R positive patients (p = 0.028). Gender was not associated with the presence of anti-PLA2R autoantibodies and the two groups were not significantly different for median serum creatinine, 24 h proteinuria and eGFR. However, serum albumin levels were significantly higher in anti-PLA2R negative patients than in anti-PLA2R positive patients (p = 0.004).

**Table 4:** Comparison of biomarkers of disease activity in anti-PLA2R autoantibody- positive and -negative patients with iMN at the time of renal biopsy

	Anti-PLA2R negative patients (n = 21)	Anti-PLA2R positive patients (n = 105)	p
Sex (male/female)	15 (71.4%)/6 (28.6%)	70 (66.6%)/35 (33.3%)	0.441
Age at diagnosis (years)^a^	52.14 ± 15.62	60.10 ± 16.99	0.028
Serum creatinine (mg/dl)^b^	4.12 (2.12–6.11)	1.09 (0.84–1.55)	0.868
Serum albumin (g/dl)^b^	3.95 (3.90–4.00)	2.20 (1.80–2.50)	0.004
Proteinuria (g/24 h)^b^	7.07 (6.38–7.75)	6.7 (4.00–9.50)	0.960
eGFR (ml/min/1.73 m^2^)^b^	40.05 (14.30–65.80)	74.00 (40.00–99.00)	0.533
Anti-PLA2R (RU/ml)^b^	2.00 (2.00–2.00)	82.00 (20.70–214.80)	0.000

*IMN* idiopathic membranous nephropathy, *eGFR* estimated glomerular

filtration rate, *PLA2R* phospholipase A2 receptor

^a^Mean ± SD

^b^Median (interquartile range)

## Discussion

The presence of serum anti-PLA2R autoantibodies has an important impact on the diagnosis of iMN, helping in differentiating it from sMN and other nephropathies [11]. In this study we evaluated the diagnostic accuracy of ELISA in detecting anti-PLA2R antibodies in a large cohort of iMN patients at the time of diagnosis before initiating immunosuppressive therapy. Studies have shown there is a very high qualitative agreement between the various serologic testing methods, all providing very high specific results [13]. Compared to cell-based indirect immunofluorescence or western blot, the ELISA has the advantage of objectivity and of quantitative measurement of anti-PLA2R antibody levels; however, the diagnostic accuracy varies according to the adopted cut-off value. The choice of the cut-off is fundamental to discriminate between positive and negative results and a large debate has arisen concerning the best cut-off to adopt in order to maximize diagnostic efficiency. If a higher cut-off is chosen, the specificity of the data is privileged, while the use of a lower cut-off favors the diagnostic sensitivity of the test.

Accordingly, in the literature, the diagnostic accuracy of PLA2R antibodies in iMN patients is variable. Referring only to the ELISA, Behnert et al. [12], using cut-offs of 14 and 20 RU/ml, in American and German cohorts of patients, reported a sensitivity of 86.1% and 82.2%, with a specificity of 84.5% and 89.7%, respectively. In a Chinese cohort, Dou et al. [20] reported that at different cut-off values of 14, 20, and 40 RU/ml, specificity did not change (97.3% for all three cut-off values), while sensitivity was 65.3%, 60.2%, and 45.8%, respectively, suggesting that 14 RU/ml should be applied to obtain higher sensitivity with no change in the specificity values. Timmermans et al. [21] suggested that sensitivity could be improved to 72% without affecting the specificity by reducing the cut-off value to 2 RU/ml (the lowest calibrator supplied with the commercially available kit). Liu et al. [22], using a cut-off value of 2.6 RU/ml, found a sensitivity of 78.9% and a specificity of 91.7% in 57 Chinese patients with iMN. Bobart et al. [23] submitted that kidney biopsy can be deferred either when ELISA is > 20 RU/ml or when ELISA ≥2 RU/ml is confirmed by indirect immunofluorescence assay. According to these authors, under these conditions it would be possible to avoid renal biopsy, especially for patients at high risk of complications or in whom a renal biopsy may be contraindicated, suggesting that the anti-PLA2R antibody could be a useful marker in non-invasive serology-based diagnosis.

To clarify this important issue and in order to harmonize anti-PLA2R antibody results (thereby correctly assessing anti-PLA2R antibody prevalence in iMN), we reviewed several studies reporting the diagnostic accuracy of anti-PLA2R antibodies using the same ELISA (the only one commercially available), with a cut-off of 20 RU/ml, or 14 RU/ml, or 2.00 and 2.7 RU/ml.

At 20 RU/ml, the average sensitivity was 65.5% with 98.4% specificity [12, 17, 20, 21, 24, 25]. At a cut-off value of 14 RU/ml, average sensitivity and specificity were 69.1% and 95.2%, respectively [12, 20, 26, 27]. Finally, in the studies in which the authors, based on ROC curves, opted for much lower cut-offs (between 2.0 and 2.7 RU/ml), the average sensitivity was 81.8% and the specificity was 93.8% [20–22, 28, 29].

Our data are in line with these figures and, in addition, some other indications come from the study. Looking at the data in Table 3 it is evident that increasing the cut-off from 14 to 20 RU/ml has no advantage; though diagnostic specificity remains the same, the slight decrease (2%) in sensitivity at 20 RU/ml reduces the LR+ and the diagnostic odds ratio. Taken together, these data show that while the commercial ELISA has the highest specificity at 14 RU/ml, optimal sensitivity is achieved at a lower cut-off, around 2.7 RU/ml which allows for a further 16% of patients to be classified. Thus, depending on the clinical need, nephrologists may use values ≥ 14 RU/ml to confirm a diagnosis of iMN and values between 2.7 and 14 RU/ml as highly suggestive for iMN, although not completely specific.

We also analyzed biomarkers of disease activity in patients with iMN: serum creatinine, serum albumin, proteinuria and eGFR. We observed that patients with anti-PLA2R negative and patients with anti-PLA2R positive differed only for serum albumin, which showed significantly higher levels in anti-PLA2R negative patients. This finding is consistent with other studies which reported a higher rate of hypoalbuminemia in anti-PLA2R positive patients [30]. On the contrary, we did not find any association of serum anti-PLA2R antibodies with proteinuria, possibly because of the milder disease course in our patient series. Indeed, it has been demonstrated that anti-PLA2R autoantibody concentrations allow assessment of disease activity earlier than proteinuria, with an increase in antibody levels preceding a rise in proteinuria and a decrease being followed by a fall in proteinuria [7].

In conclusion, this study showed that although the best cut-off for the widely used ELISA method to detect anti-PLA2R antibodies in iMN patients is probably much lower (around 2–2,7 RU/ml) than that indicated by the manufacturer, thereby relevantly increasing assay sensitivity, the cut-off that guarantees the best clinical performance represented by positive likelihood ratio and diagnostic odds ratio could be positioned at 14 RU/ml. Depending on the clinical use (early diagnosis or as a support to confirm clinical diagnosis), nephrologists may take advantage of this evidence by choosing the most convenient option. However, renal biopsy remains mandatory for the definitive diagnosis of iMN and assessment of disease severity.
